# Deutsche Übersetzung und Validierung des VMIQ-2 zur Erfassung der Lebhaftigkeit von Handlungsvorstellungen

**DOI:** 10.1026/1612-5010/a000273

**Published:** 2019-12-17

**Authors:** Stephan F. Dahm, Victoria K. E. Bart, Jan M. Pithan, Martina Rieger

**Affiliations:** 1 UMIT – Private Universität für Gesundheitswissenschaften, Medizinische Informatik und Technik, Hall in Tirol; 2 Martin-Luther-Universität Halle-Wittenberg

**Keywords:** Motor imagery, external visual imagery, internal visual imagery, kinesthetic imagery, questionnaire

## Abstract

The aim of the study was to validate a German version of the Vividness of Movement Imagery Questionnaire 2 (VMIQ-2; Roberts, Callow, Hardy, Markland, & Bringer, 2008), which measures external visual, internal visual, and kinesthetic vividness of movement imagery. The psychometric characteristics of the German version did not differ significantly from the English version. Using confirmatory factor analyses, the three-dimensional structure of the VMIQ-2 was replicated with reasonable fit and good internal consistency. The test-retest reliability was moderate. Thus, the German version of the VMIQ-2 is a valid instrument for measuring the vividness of movement imagery.

Handlungsvorstellungen bezeichnen mentale Repräsentationen einer Handlung, ohne die Handlung tatsächlich auszuführen (Jeannerod, 1994). Beispielsweise kann man sich vorstellen im Meer zu schwimmen und dabei die Wellen sehen, die Bewegungen der Arme und Beine spüren, die Temperatur des Wassers fühlen, das Salz im Wasser schmecken und den Duft des Meeres riechen. Prinzipiell können Handlungsvorstellungen alle Modalitäten (visuell, kinästhetisch, taktil, akustisch, olfaktorisch, gustatorisch) beinhalten (Cumming & Eaves, 2018). Der visuellen und kinästhetischen Modalität von Handlungsvorstellungen wird jedoch in der Forschung oftmals mehr Bedeutung zugeschrieben (Jeannerod, 1994; Roberts, Callow, Hardy, Markland & Bringer, 2008; Williams et al., 2012). Dies liegt vermutlich darin begründet, dass visuelle und kinästhetische Aspekte, im Gegensatz zu anderen Modalitäten, bei fast allen Handlungen von Bedeutung sind. Beispiels-weise treten gustatorische Vorstellungen vermutlich auf, wenn man sich vorstellt ein Kaugummi zu kauen, jedoch eher nicht, wenn man sich vorstellt Fahrrad zu fahren. Darüber hinaus hat sich historisch gesehen die Forschung zu Handlungsvorstellungen (Jeannerod, 1994) aus dem Forschungsfeld zu visuellen Vorstellungen (Marks, 1973) entwickelt. Im Laufe der Zeit wurde die Bedeutung kinästhetischer Aspekte für Handlungsvorstellungen immer deutlicher (Isaac, Marks & Russell, 1986).

Visuelle Vorstellungen können aus externaler oder internaler Perspektive generiert werden (Roberts et al., 2008). In der externalen Perspektive, auch dritte Person Perspektive, betrachtet man sich selbst von außen, wie auf einem Video. In der internalen Perspektive, auch erste Person Perspektive, erfolgt die Vorstellung aus der eigenen Sicht, wie durch die eigenen Augen. Kinästhetische Vorstellungen erfolgen in der internalen Perspektive und beinhalten das mit der Handlung verbundene Körpergefühl. Gelegentlich wird in der Literatur zu Handlungsvorstellungen in der internalen Perspektive nicht zwischen internal visuellen und kinästhetische Handlungsvorstellungen differenziert (Isaac et al., 1986). Handlungsvorstellungen aus der internalen Perspektive implizieren jedoch nicht automatisch eine Kombination der visuellen und kinästhetischen Modalitäten (Roberts et al., 2008). Stattdessen scheinen sich Personen in ihrer Präferenz für Modalitäten und Perspektiven zu unterscheiden (Moran, Campbell, Holmes & MacIntyre, 2012).

Des Weiteren unterscheiden sich Personen auch in ihrer Handlungsvorstellungsfähigkeit (Cumming & Eaves, 2018). Die Handlungsvorstellungsfähigkeit beschreibt die Fähigkeit, lebhafte und kontrollierbare Vorstellungen zu generieren und für längere Zeit aufrechtzuerhalten (Morris, Spittle & Watt, 2005). Sie beinhaltet dabei verschiedene Dimensionen wie die Generierbarkeit, die Lebhaftigkeit, die Persistenz und die Kontrollierbarkeit von Handlungsvorstellungen (Cumming & Eaves, 2018). Die Generierbarkeit beschreibt, wie leicht Handlungsvorstellungen erzeugt werden können (Williams et al., 2012). Die Lebhaftigkeit beschreibt, wie intensiv Handlungsvorstellungen wahrgenommen werden (Roberts et al., 2008). Die Persistenz beschreibt, ob die Handlungsvorstellungen über längere Zeit aufrechterhalten werden können (Madan & Singhal, 2013). Die Kontrollierbarkeit beschreibt, inwiefern Handlungvorstellungen intentional manipuliert werden können (Cumming & Eaves, 2018), d. h. ob beispielsweise die Geschwindigkeit der Handlung in der Vorstellung intentional verändert werden kann (Guillot & Collet, 2005). Zudem wird die Handlungsvorstellungsfähigkeit auch davon beeinflusst, welche sensorischen Modalitäten (auditiv, visuell, kinästhetisch) für die Handlungsvorstellung benutzt werden, wobei sich die Modalitäten auch wechselseitig verstärken könnten (Lacey & Lawson, 2013).

Zur Erfassung der Dimensionen von Handlungsvorstellungsfähigkeit gibt es bereits einige Fragebögen, welche die verschiedenen Perspektiven und Modalitäten berücksichtigen (für einen Überblick siehe Pithan & Dahm, 2015). Beispielsweise wird die Generierbarkeit von Handlungsvorstellungen im Movement Imagery Questionnaire (MIQ-3; Williams et al., 2012) und im Sport Imagery Ability Questionnaire erfasst (SIAQ; Williams & Cumming, 2011; deutsche Version: Simonsmeier & Hannemann, 2017). Eine andere Dimension, die Lebhaftigkeit von Handlungsvorstellungen, wird mit dem Vividness of Movement Imagery Questionnaire (VMIQ; Isaac et al., 1986) und dessen Revision (VMIQ-2; Roberts et al., 2008) gemessen.

Die Lebhaftigkeit wird im VMIQ-2 erfasst, indem 12 verschiedene Handlungen jeweils internal visuell, external visuell und kinästhetisch vorgestellt werden. Im Anschluss daran wird die Lebhaftigkeit der Vorstellung beurteilt (Roberts et al., 2008). Die Validierung des VMIQ-2 bestätigte die angenommene dreifaktorielle Struktur (internal visuell, external visuell und kinästhetisch) und zeigte gute psychometrische Kennwerte sowie eine gute Konstruktvalidität (Roberts et al., 2008). Der VMIQ-2 wird daher häufig in Experimenten zu Handlungsvorstellungen verwendet, um Versuchspersonen auszuschließen, die sich Handlungen nicht oder wenig lebhaft vorstellen können (Callow, Roberts, Hardy, Jiang & Edwards, 2013; O’Shea & Moran, 2019). Der Fragebogen ist zudem geeignet, um zwischen Personen mit internalen und externalen Vorstel-lungspräferenzen zu unterscheiden (Dana & Gozalzadeh, 2017). Außerdem wird er eingesetzt, um Veränderungen in der Lebhaftigkeit von Handlungsvorstellungen zu untersuchen. So zeigte sich beispielsweise, dass Handlungsvorstellungstraining insbesondere die Lebhaftigkeit kinästhetischer Vorstellungen verbessert (Abraham, Gose, Schindler, Nelson & Hackney, 2019). Des Weiteren ist die Erfassung der Lebhaftigkeit von Handlungsvorstellungen auch für die sportpsychologische Anwendung relevant, insbesondere für mentales Training, d. h. der wiederholten und planmäßigen Durchführung von Handlungsvorstellungen mit dem Ziel, die Handlungsausführung zu verbessern (Driskell, Copper & Moran, 1994). Beispielsweise verbessern sich Personen mit lebhaften Handlungsvorstellungen mehr durch mentales Training in der Ausführung von Trampolinsprüngen als Personen mit weniger lebhaften Handlungsvorstellungen (Isaac, 1992).

Obwohl die Erfassung der Lebhaftigkeit von Handlungsvorstellungen in der Wissenschaft und der sportpsychologische Anwendung an Bedeutung gewinnt, gibt es bislang keine deutschsprachige Übersetzung des VMIQ-2. Daher war das Ziel dieser Arbeit, den VMIQ-2 zu übersetzen und die psychometrischen Eigenschaften der deutschen Übersetzung zu prüfen.

## Methode

### Stichprobe

Die minimale Stichprobengröße wurde basierend auf der niedrigsten Faktorladung in der englischsprachigen Version des VMIQ-2 (a = .6; Roberts et al., 2008) und der Annahme von 3 Faktoren geschätzt. Dies resultierte in einer geschätzten Stichprobengröße von 220 Versuchspersonen (Wolf, Harrington, Clark & Miller, 2013). Untersucht wurden 254 deutschsprachige Studierende (*M*_Alter_ = 24.0, *SD* = 5.0 Jahre, 175 Frauen), welche freiwillig teilnahmen, über die Studie aufgeklärt wurden und schriftlich ihr Einverständnis zur Teilnahme gaben. Eine Teilstichprobe (*N* = 78, *M*_Alter_ = 24.0, *SD* = 3.5 Jahre, 48 Frauen) füllte den Fragebogen nach 6 Wochen erneut aus. Der Fragebogen wurde im Kontext verschiedener Experimente miterhoben. Die Studierenden erhielten Versuchspersonenstunden für die Teilnahme.

### Material und Durchführung

Der VMIQ-2 besteht aus 3 Skalen mit je 12 Items. Die 12 Items beinhalten alltägliche Handlungen (Gehen, Rennen), Handlungen, die Präzision voraussetzen (einen Stein wegkicken, sich Bücken um eine Münze aufzuheben), Handlungen, bei denen man ein Hindernis überwindet (eine Treppe hoch rennen, zur Seite springen), Handlungen, bei denen ein Objekt manipuliert wird (einen Stein ins Wasser werfen, einen Ball in die Luft schießen), schnelle Handlungen, die Gleichgewicht erfordern (bergab rennen, Fahrrad fahren), und Handlungen, bei denen Objekte / Bewegungen in der Luft kontrolliert werden (ein Seil schwingen, eine hohe Mauer herunterspringen; Isaac et al., 1986). Jede Handlung wird mit geschlossenen Augen jeweils external visuell (EVI), internal visuell (IVI) sowie kinästhetisch (KIN) vorgestellt. Bei external visuellen Handlungsvorstellungen werden die Personen aufgefordert, sich in ihrer Vorstellung wie auf einem Video bei der Bewegungsausführung zu sehen. Bei internal visuellen Handlungsvorstellungen werden die Personen aufgefordert, sich in ihrer Vorstellung wie durch ihre eigenen Augen zu sehen. Bei kinästhetischen Handlungsvorstellungen werden die Personen aufgefordert, sich in die Bewegung hinein zu fühlen und sich vorzustellen was sie während der Bewegung fühlen. Im Anschluss an die Vorstellung wird die Lebhaftigkeit der Vorstellung auf einer Ratingskala bewertet (1 – absolut klar und deutlich wie in Wirklichkeit, 2 – klar und einigermaßen lebhaft, 3 – mittelmäßig klar und lebhaft, 4 – vage und unklar, 5 – keine Vorstellung, ich weiß lediglich, dass ich an die Bewegung denke).

Die Übersetzung des VMIQ-2 folgte einem interaktiven Ansatz (Douglas & Craig, 2007) mit zunächst unabhängigen Übersetzungen von zwei Personen mit Fachexpertise (Epstein, Osborne, Elsworth, Beaton & Guillemin, 2015). Abweichungen in deren Übersetzung wurden anschließend gemeinsam besprochen und der Fragebogen wurde entsprechend überarbeitet. Anschließend wurde der Fragebogen an einzelnen Versuchspersonen getestet, die die Inhalte des Fragebogens in eigenen Worten wiedergeben sollten, um Formulierungen zu optimieren (Douglas & Craig, 2007). Die überarbeitete Version wurde letztendlich von einer dritten Person geprüft. Abweichungen von einer wortgetreuen Übersetzung sind im Anhang A dargestellt und begründet. Alle Personen, die an der Übersetzung beteiligt waren, haben ausgewiesene Expertise in der englischen und deutschen Sprache sowie auf dem Gebiet der Handlungsvorstellungen, u. a. ersichtlich durch facheinschlägige Publikationen in international anerkannten Fachzeitschriften. Ein PDF des deutschsprachigen Fragebogens ist im Zusatzmaterial bereitgestellt.

## Ergebnisse

Zum Vergleich der deutschen und englischen Version wurden die Originaldaten^1^ (Roberts et al., 2008) verwendet. Als Kennwerte der Faktoren sind Mittelwerte, Standardabweichungen, Schiefe, Kurtosis, Cronbach’s α und größte und kleinste Faktorladung in Tabelle 1 dargestellt. Eine vollständige Übersicht der Faktorladungen findet sich im Anhang B.

Konfirmatorische Faktorenanalysen mit Satorra-Bentler Korrektur unter der Annahme von 3 Faktoren (EVI, IVI, KIN) mit jeweils 12 Items wurden mit dem R Paket „lavaan“ (Rosseel, 2012) berechnet. Analog zu Roberts et al. (2008) wurden zur Beurteilung der Modellpassung folgende Fit-Indizes berechnet: Satorra-Bentler X^2^ Statistik (S-B X^2^), das Verhältnis von X^2^ Werten zu Freiheitsgraden (X^2^ / *df*), der comparative fit index (CFI), der standardized root mean square residual (SRMR) und der root mean square error of approximation (RMSEA). Eine gute Modellpassung wird angenommen bei a) einem Verhältnis von X^2^ Werten zu Freiheitsgraden, das kleiner als 3:1 ist, b) einem CFI von größer .9, c) einem SRMR von kleiner als .1, und d) einem *RMSEA* kleiner als .05 (Hair et al., 2014).

Analog zu Roberts et al. (2008) wurde ein Multi-Trait-Multi-Method Ansatz (MTMM) unter Verwendung eines correlated trait – correlated uniqueness Modells (CTCU, Kenny, 1976) genutzt. Der MTMM Ansatz berücksichtigt, dass es sowohl für die Items eines Faktors als auch für die Items einer Handlung einen gemeinsamen Varianzanteil gibt. Das geschieht im CTCU Modell, indem die Faktoren korreliert werden und die gemeinsamen Varianzanteile der einzelnen Handlungen aus den unsystematischen Fehlervarianzen der einzelnen Items herausgerechnet werden. Zusätzlich wurde ein Modell mit dem Multi-Trait (MT) Ansatz berechnet, bei dem allein die Faktoren (EVI, IVI, KIN) einbezogen wurden, ohne die Kovarianzen gleicher Handlungen zu berücksichtigen. Die Fit-Indizes der beiden Ansätze sind in Tabelle 2 dargestellt. Nur die Fit-Indizes des MTMM Ansatzes weisen auf eine akzeptable Modellpassung hin (Hair et al., 2014). Mittels Satorra-Bentler X^2^ Unterschiedstests wurden der MTMM Ansatz und der MT Ansatz hinsichtlich ihrer Modellpassung verglichen (Satorra & Bentler, 2001). Die Modellpassung war im MTMM Ansatz signifikant besser als im MT Ansatz (*p* < .001).

Die Fit-Indizes der englischen Version (Roberts et al., 2008) wurden neu berechnet (Tab. 2), da die aktuelle Statistiksoftware (LISREL 9, lavaan) andere Berechnungsgrundlagen verwendet. Nur so konnte eine Vergleichbarkeit der Modellpassung der englischen und der deutschen Version gewährleistet werden. Die Modellpassung der deutschen und englischen Version wurde ebenfalls mittels Satorra-Bentler X^2^ Unterschiedstest verglichen (Satorra & Bentler, 2001). Die Modellpassung der englischen und deutschen Version unter Berücksichtigung des MTMM Ansatzes unterschieden sich nicht signifikant (*p* > .99).

Analog zu Roberts et al. (2008) wurden Interfaktor-Korrelationen zwischen EVI, IVI und KIN berechnet (Tab. 3). Zum Vergleich der Interfaktor-Korrelationen der englischen und deutschen Version wurden Fishers z Werte berechnet. Die Korrelationen zwischen KIN und den visuellen Vorstellungen (IVI und EVI) unterschieden sich nicht signifikant zwischen deutscher und englischer Version.

Die Korrelation zwischen den visuellen Vorstellungsperspektiven EVI und IVI war in der deutschen Version signifikant höher als in der englischen Version. Daher wurden zwei Modelle verglichen, die EVI und IVI entweder als separate Faktoren oder als gemeinsamen Faktor betrachten. Ein Satorra-Bentler X^2^ Unterschiedstest (Satorra & Bentler, 2001) ergab, dass die Modellpassung des Zwei-Faktorenmodells, Satorra-Bentler X^2^(239) = 416, X^2^ / *df* = 1.74, *CFI* = 0.93, *SRMR* = 0.06, *RMSEA* = 0.05, signifikant besser war (*p* < .001) als die des Ein-Faktormodells, Satorra-Bentler X^2^(240) = 947, X^2^ / *df* = 3.95, *CFI* = 0.71; *SRMR* = 0.11, *RMSEA* = 0.11.

Da die Faktoren IVI und KIN in der Literatur teilweise nicht getrennt werden (Isaac et al., 1986), wurden angelehnt an Roberts et al. (2008) zwei weitere Modelle miteinander verglichen, die IVI und KIN als separate Faktoren oder als gemeinsamen Faktor betrachten. Das Zwei-Faktorenmodell beinhaltete zwei separate Faktoren IVI und KIN, Satorra-Bentler X^2^(239) = 496, X^2^ / *df* = 2.07, *CFI* = 0.89; *SRMR* = 0.06, *RMSEA* = 0.07. Im Ein-Faktorenmodell wurden IVI und KIN als ein Faktor betrachtet, indem die Korrelation auf 1 gesetzt wurde, Satorra-Bentler X^2^(240) = 1015, X^2^ / *df* = 4.23, *CFI* = 0.66; *SRMR* = 0.1, *RMSEA* = 0.11. Ein Satorra-Bentler X^2^ Unterschiedstest (Satorra & Bentler, 2001) ergab, dass die Modellpassung für das Zwei-Faktorenmodell signifikant besser war als die des Ein-Faktormodells, *p* < .001.

Zur Messung der Test-Retest-Reliabilität wurde der Konkordanz-Korrelations-Koeffizient berechnet (Lin, 1989). Die Konkordanz-Korrelations-Koeffizienten betrugen *r*_tt_ = .62 für EVI, *r*_tt_ = .61 für IVI, *r*_tt_ = .64 für KIN und *r*_tt_ = .69 für den VMIQ-2-Gesamtwert der deutschen Version.

## Diskussion

Der VMIQ-2 (Roberts et al., 2008) wurde ins Deutsche übersetzt und die psychometrischen Eigenschaften der Übersetzung wurden analysiert. Die erwartete dreidimensionale Struktur des VMIQ-2 konnte repliziert werden. Das Verhältnis von X^2^ Werten zu Freiheitsgraden (1.6) war kleiner als 3, CFI (.92) war größer als .9, SRMR (.06) war kleiner als .1, und RMSEA genau .05. Entsprechend sind die Fit-Indizes des MTMM Ansatzes der deutschen Version als akzeptabel zu bewerten (Hair et al., 2014). Die Modellpassung der deutschen Version unterschied sich nicht signifikant von der Modellpassung der englischen Version (Roberts et al., 2008). Dies spricht für eine gelungene Übersetzung des Fragebogens. Jeder der drei Faktoren hat eine hohe interne Konsistenz (über α > .7; Hair et al., 2014), die mit der englischen Version vergleichbarist. Die Werte der Test-Retest-Reliabilität waren moderat (.5 < *r*_tt_ < .75; Koo & Li, 2016).

Der MTMM Ansatz erbrachte eine bessere Modellpassung als ein Modell ohne Multi-Methoden Ansatz. Dies spricht dafür, dass sowohl die Items eines Faktors als auch die Items einer Handlung einen gemeinsamen Varianzanteil haben (Roberts et al., 2008). Die mittleren Korrelationen zwischen den Faktoren bestätigen die Annahme, dass die einzelnen Faktoren Unterkategorien des Generalfaktors Handlungsvorstellung sind (Roberts et al., 2008). Dennoch konnte wie auch in der englischen Version gezeigt werden, dass IVI und KIN als separate Faktoren zu betrachten sind. In gleicher Weise sind IVI und EVI als separate Faktoren zu betrachten.

Die Stichprobenzusammensetzung zur Validierung des VMIQ-2 unterschied sich in der deutschen und englischen Version nicht nur sprachlich-kulturell. Der deutsche Fragebogen wurde an Studierenden validiert, während der englische Fragebogen an Athletinnen und Athleten validiert wurde. Zudem waren in der deutschen Stichprobe prozentual mehr Frauen als in der englischen Stichprobe. Verschiedene Subpopulationen könnten sich in der Lebhaftigkeit von Handlungsvorstellungen unterscheiden. Beispielsweise haben Top-Athletinnen und Top-Athleten oftmals lebhaftere Handlungsvorstellungen als Amateursportlerinnen und Amateursportler (Roberts et al., 2008). Die Faktorstruktur eines Fragebogens sollte jedoch für alle Subpopulationen gleich sein. Tatsächlich ließ sich die Faktorstruktur der englischen Version mit der deutschen Version replizieren. Zusätzlich unterschied sich die Passung beider Versionen nicht signifikant.

Mittelwerte, Streuung und Schiefe der einzelnen Faktoren beider Versionen lassen Deckeneffekte erkennen. Es ist auffallend, dass die Teilnehmenden selten eine unklare Handlungsvorstellung berichteten. Die Ursache hierfür könnte in einer Tendenz zu einer positiven Selbstdarstellung liegen. Verzerrungen durch soziale Erwünschtheit haben auf Fragebögen einen stärkeren Einfluss als auf Tests (Nederhof, 1985). Entsprechend lassen sich Defizite der Handlungsvorstellungsfähigkeit möglicherweise besser durch Tests anstelle von Fragebögen aufdecken (Dahm, 2019; Morris et al., 2005). Im Rahmen eines solchen Tests werden Personen beispielsweise aufgefordert sich eine Reihe von Bewegungen nacheinander vorzustellen und anschließend anzugeben, in welcher Endposition sich ihr Körper befindet (Madan & Singhal, 2013). Dies sollte Personen mit hoher Handlungsvorstellungsfähigkeit besser gelingen. Eine alternative Erklärung für die beobachteten Deckeneffekte wäre, dass es sich bei den Handlungen des VMIQ-2 um einfache Alltagshandlungen handelt, bei denen es möglicherweise fast jeder Person leicht fällt sich diese lebhaft vorzustellen, da sie mit ihnen vertraut sind.

Die Tatsache, dass beim VMIQ-2 vor allem einfache und alltägliche Handlungen vorgestellt werden, könnte den Transfer für die Anwendung, z. B. im Leistungssport, einschränken. Möglicherweise unterscheidet sich die Lebhaftigkeit von Handlungsvorstellungen zwischen einfachen und komplexen Handlungen. Auch könnte die Lebhaftigkeit von Handlungsvorstellungen von der Handlungsexpertise einer Person abhängen. Zwei Personen mit unterschiedlichem Wissen über einen Tennisaufschlag könnten zwar eine ähnliche generelle Handlungsvorstellungsfähigkeit haben, sich aber in der Handlungsvorstellungsfähigkeit des Tennisaufschlages unterscheiden (Schack, Essig, Frank & Koester, 2014). Dafür spricht, dass die Abfolge einzelner Elemente einer Handlung bei Ex-perten stärker in einer hierarchischen Struktur repräsentiert ist als bei Novizen (Schack & Mechsner, 2006). Außerdem stimmen die Dauer von Handlungsvorstellung und Handlungsausführung bei vertrauten Handlungen besser überein als bei unvertrauten Handlungen (Rieger, 2012). Auch die bessere Passung des MTMM Ansatzes im Vergleich zu dem MT Ansatz, spricht für die Annahme, dass Vorstellungen handlungsspezifisch sind. Je nach Einsatzzweck könnte es demnach sinnvoll sein, die Lebhaftigkeit von Handlungsvorstellungen zusätzlich zum VMIQ-2 mit spezifischen Fragen für die untersuchte Handlung zu ergänzen.

Die Test-Retest Reliabilität des VMIQ-2-Gesamtwertes ist mit *r*_tt_ = .69 als moderat einzustufen und liegt damit knapp unter dem Referenzwert einer guten Test-Retest Reliabilität von *r*_tt_ = .75 (Koo & Li, 2016). Deckeneffekte könnten hier eine Rolle gespielt haben, da eine geringe Varianz mit niedrigeren Korrelationen einhergeht. Es ist ebenso möglich, dass die Lebhaftigkeit von Handlungsvorstellungen veränderbar ist (Abraham et al., 2019). Für den VMIQ-2 (Roberts et al., 2008) lagen bislang keine Test-Retest Reliabilitätswerte vor.

Fragebögen allein erfassen die Handlungsvorstellungsfähigkeit nicht zur Gänze, sondern nur spezifische Aspekte, so wie der VMIQ-2 nur die Lebhaftigkeit von Handlungsvorstellungen erfasst. Die Messung der Handlungsvorstellungsfähigkeit anhand von Fragebögen weist manchmal keinen Zusammenhang mit dem Vergleich von Vorstellungs- und Ausführungsdauern (mentale Chronometrie) auf, selbst wenn für letzteres die Handlungen aus dem Fragebogen verwendet werden (Williams, Guillot, Di Rienzo & Cumming, 2015). Dies deutet darauf hin, dass Fragebögen und mentale Chronometrie andere, voneinander unabhängige Dimensionen von Handlungsvorstellungen erfassen. Beispielsweise könnten Fragebögen eher die Fähigkeit erfassen lebhafte Vorstellungen zu generieren, während die Übereinstimmung von Vorstellungs- und Ausführungsdauer eher davon beeinflusst wird, wie gut es Personen gelingt die Vorstellung aufrechtzuerhalten und zu kontrollieren (Williams et al., 2015). Zukünftige Arbeiten sollten auf die Entwicklung weiterer Verfahren zur Erfassung unterschiedlicher Aspekte von Handlungsvorstellungen setzen (Dahm, 2019). Dies würde auch ermöglichen die Konstruktvalidität der deutschen Version des VMIQ-2 genauer zu evaluieren.

Zusammenfassend zeigt sich, dass die deutsche Version des VMIQ-2 gute psychometrische Kennwerte und akzeptable Fit-Indizes aufweist und der dreidimensionalen Struktur der englischen Version entspricht. Die deutsche Version stellt daher ein valides deutschsprachiges Messinstrument zur Erfassung der Lebhaftigkeit von internal visuellen, external visuellen und kinästhetischen Handlungsvorstellungen für den deutschsprachigen Raum dar.

## Supplementary Material

Die elektronischen Supplemente sind mit der Online-Version dieses Artikels verfügbar unter https://doi.org/1612-5010/a000273

**ESM 1.** Fragebogen VMIQ-2 (deutsche Version)

Fragebogen

Anhang

## Figures and Tables

**Tabelle 1:** Kennwerte der Faktoren (EVI = external visuell, IVI = internal visuell, KIN = kinästhetisch) der deutschen und englischen Version des VMIQ-2

	VMIQ-2-Deutsch (*N* = 254)			VMIQ-2 Englisch (*N* = 351)		
	EVI	IVI	KIN	EVI	IVI	KIN
*M*	2	1.8	2	2.4	2.1	2.2
*SD*	0.7	0.6	0.7	0.8	0.8	0.8
Schiefe	0.6	0.9	0.8	0.6	0.9	0.8
Kurtosis	0.2	0.7	0.8	0.3	0.6	0.9
Cronbach’s α	.91	.9	.91	.93	.93	.92
a_min_	.57	.56	.6	.68	.66	.6
a_max_	.75	.73	.74	.78	.78	.78

*Anmerkungen:* a_min_ = kleinste Faktorladung, a_max_ = größte Faktorladung

**Tabelle 2:** Fit-Indizes der konfirmatorischen Faktorenanalysen der englischen und deutschen Version des VMIQ-2

	S-B X^2^ (*df*)	X^2^/*df*	*CFI*	*SRMR*	*RMSEA*
Englisch MTMM	857 (555)	1.54	.95	.04	.039
Deutsch MTMM	903 (555)	1.63	.92	.06	.05
Deutsch MT	1693 (591)	3.05	.74	.08	.082

*Anmerkungen:* MTMM = Multi-Trait-Multi-Methoden Ansatz, MT = Multi-Trait Ansatz

**Tabelle 3:** Vergleich von Korrelationen in der deutschen und der englischen Version des VMIQ-2 zwischen external visuellen (EVI), internal visuellen (IVI) und kinästhetischen (KIN) Handlungsvorstellungen

	Deutsch	Englisch	*Z*	*p*
EVI x IVI	.53	.39	2.2	.016
EVI x KIN	.44	.41	0.4	.33
IVI x KIN	.59	.63	0.8	.22

## Footnotes

1 Zur Verfügung gestellt von Ross Roberts.
