# Supplementary material for: Deutsche Übersetzung und Validierung des VMIQ-2 zur Erfassung der Lebhaftigkeit von Handlungsvorstellungen
Source: Z Sportpsychol. Author manuscript; Available in PMC 2020 Apr 9. (PMC7145442; doi:10.1026/1612-5010/a000273)
Supplement: Fragebogen [file EMS85440-supplement-Fragebogen.pdf]

VMIQ-2 Datum: VPNR:

Auf den nächsten Seiten werden Ihnen einige Fragen zur Bewegungsvorstellung gestellt. Dieser Fragebogen dient dem Zweck zu messen, wie lebhaft Ihre Vorstellungen von Bewegungen sind. In dem Fragebogen werden verschiedene Bewegungen beschrieben. Diese sollen bestimmte Bilder in Ihrer Vorstellung hervorrufen. Sie werden gebeten, die Lebhaftigkeit einer jeden Vorstellung auf einer 5-stufigen Skala zu bewerten, indem Sie die entsprechende Zahl ankreuzen. Zunächst werden Sie gebeten, sich jede Bewegung aus einer externen visuellen Perspektive vorzustellen. Das ist vergleichbar mit einem Video auf dem Sie sich selbst sehen wie Sie die Bewegung ausführen. Anschließend werden Sie gebeten, sich jede Bewegung aus einer internen visuellen Perspektive vorzustellen. Stellen Sie sich vor, Sie schauen durch Ihre eigenen Augen, während Sie die Bewegung ausführen. Abschließend werden Sie gebeten, sich in jede Bewegung hineinzufühlen. Stellen Sie sich vor, was Sie während der Bewegung fühlen (kinästhetische Vorstellung). Versuchen Sie, jede Bewertung unabhängig von den anderen durchzuführen. Die drei verschiedenen Bewertungen pro Bewegung müssen dementsprechend nicht identisch sein. Gehen Sie bei jeder Bewegung wie folgt vor: Lesen Sie die Bewegung, schließen Sie die Augen, stellen Sie sich die Bewegung vor, bewerten Sie die Lebhaftigkeit der Bewegung.

Beispiel

Absolut klar und deutlich wie in Wirklichkeit	Klar und einigermaßen lebhaft	Mäßig klar und lebhaft	Vage und unklar	Keine Vorstellung, ich weiß lediglich, dass ich an die Bewegung denke
1	2	3	4	5

Referenzen

Roberts, R., Callow, N., Hardy, L., Markland, D. & Bringer, J. (2008). Movement imagery ability: development and assessment of a revised version of the vividness of movement imagery questionnaire. *Journal of Sport and Exercise Psychology, 30*(2), 200-221.

Aus dem englischen übersetzt und validiert von Stephan F. Dahm, Victoria K.E. Bart, Jan M. Pithan und Martina Rieger. Die Publikation ist zu finden in der Zeitschrift für Sportpsychologie: ‚Deutsche Übersetzung und Validierung des VMIQ-2 zur Erfassung der Lebhaftigkeit von Handlungsvorstellungen‘

	**Sie sehen sich wie auf einem Video bei der Bewegungsausführung (extern visuelle Vorstellung)**				
Bewegung	Absolut klar und deutlich wie in Wirklichkeit	Klar und einigermaßen lebhaft	Mittelmäßig klar und lebhaft	Vage und unklar	Keine Vorstellung, ich weiß lediglich, dass ich an die Bewegung denke
1.Gehen	1	2	3	4	5
2.Rennen	1	2	3	4	5
3.Einen Stein wegkicken	1	2	3	4	5
4.Bücken um eine Münze aufzuheben	1	2	3	4	5
5.Treppe hoch rennen	1	2	3	4	5
6.zur Seite Springen	1	2	3	4	5
7.Einen Stein ins Wasser werfen	1	2	3	4	5
8.Einen Ball in die Luft schießen	1	2	3	4	5
9.Bergab rennen	1	2	3	4	5
10.Fahrrad fahren	1	2	3	4	5
11.Ein Seil schwingen	1	2	3	4	5
12.Eine hohe Mauer herunter springen	1	2	3	4	5

	**Sie sehen bei der Bewegungsausführung durch Ihre eigenen Augen (intern visuelle Vorstellung)**				
Bewegung	Absolut klar und deutlich wie in Wirklichkeit	Klar und einigermaßen lebhaft	Mittelmäßig klar und lebhaft	Vage und unklar	Keine Vorstellung, ich weiß lediglich, dass ich an die Bewegung denke
1.Gehen	1	2	3	4	5
2.Rennen	1	2	3	4	5
3.Einen Stein wegkicken	1	2	3	4	5
4.Bücken um eine Münze aufzuheben	1	2	3	4	5
5.Treppe hoch rennen	1	2	3	4	5
6.zur Seite Springen	1	2	3	4	5
7.Einen Stein ins Wasser werfen	1	2	3	4	5
8.Einen Ball in die Luft schießen	1	2	3	4	5
9.Bergab rennen	1	2	3	4	5
10.Fahrrad fahren	1	2	3	4	5
11.Ein Seil schwingen	1	2	3	4	5
12.Eine hohe Mauer herunter springen	1	2	3	4	5

VMIQ-2 **Datum:** **VPNR:**

	**Sie fühlen wie Sie die Bewegung ausführen (kinästhetische Vorstellung)**				
Bewegung	Absolut klar und deutlich wie in Wirklichkeit	Klar und einigermaßen lebhaft	Mittelmäßig klar und lebhaft	Vage und unklar	Keine Vorstellung, ich weiß lediglich, dass ich an die Bewegung denke
1.Gehen	1	2	3	4	5
2.Rennen	1	2	3	4	5
3.Einen Stein wegkicken	1	2	3	4	5
4.Bücken um eine Münze aufzuheben	1	2	3	4	5
5.Treppe hoch rennen	1	2	3	4	5
6.zur Seite Springen	1	2	3	4	5
7.Einen Stein ins Wasser werfen	1	2	3	4	5
8.Einen Ball in die Luft schießen	1	2	3	4	5
9.Bergab rennen	1	2	3	4	5
10.Fahrrad fahren	1	2	3	4	5
11.Ein Seil schwingen	1	2	3	4	5
12.Eine hohe Mauer herunter springen	1	2	3	4	5
