# Supplementary material for: Deutsche Übersetzung und Validierung des VMIQ-2 zur Erfassung der Lebhaftigkeit von Handlungsvorstellungen
Source: Z Sportpsychol. Author manuscript; Available in PMC 2020 Apr 9. (PMC7145442; doi:10.1026/1612-5010/a000273)
Supplement: Anhang [file EMS85440-supplement-Anhang.pdf]

https://econtent.hogrefe.com/doi/pdf/10.1026/1612-5010/a000273 - Tuesday, December 17, 2019 7:09:27 AM - UMIT-Priv. Universität für Gesundheitswissenschaften, Med. Informatik & Technik IP Address:193.170.165.4

Anhang A

Abweichungen der deutschen von der englischen Version

Anstelle einer wortgetreuen Übersetzung haben wir bei der Benennung der Skalen-Stufen eine freie Übersetzung vorgezogen, die der Allgemeinverständlichkeit dienen soll.

1. Englisch: "*No image at all, you only know that you are thinking of the skill*"
   Deutsch: „*Keine Vorstellung, ich weiß lediglich, dass ich an die Bewegung denke*"
   a) Statt dem Englischen „you", haben wir „ich" gewählt. Dies kommt in psychologischen Fragebögen häufiger zur Anwendung. Bei einer wörtlichen Übersetzung hätten wir uns zwischen dem förmlichen „Sie" und dem informellen „Du" entscheiden müssen. Der Fragebogen soll aber sowohl in informelleren Kontexten, wie z.B. im Sport, als auch in formelleren Kontexten verwendet werden.
   b) Da es in der deutschen Alltagssprache außerhalb der Wissenschaft eher unüblich ist, bei einigen der beschriebenen Handlungen von Fertigkeiten (skill) zu sprechen, haben wir diesen Begriff durch ‚Bewegung' ersetzt. Beispielsweise wäre es im Alltag eher nicht üblich, die Handlung, sich zu Bücken um eine Münze aufzuheben, als Fertigkeit zu bezeichnen.

2. Englisch (EVI, IVI): "*Perfectly clear and as vivid as normal vision*"
   Englisch (KIN): "*Perfectly clear and as vivid as normal feel of movement*"
   Deutsch (EVI, IVI, KIN): „*Absolut klar und deutlich wie in Wirklichkeit*"
   a) Wir haben die Benennung der Skalen-Stufen angepasst, um für alle drei Faktoren (EVI, IVI, KIN) die gleiche Formulierung zu verwenden.
   b) In einer ersten Version haben wir die Formulierung „wie normalerweise" verwendet. Einige Versuchspersonen, die den Fragebogen in eigenen Worten wiedergeben sollten und die wir zur Allgemeinverständlichkeit des Fragebogens befragt haben, meinten, dass unklar bliebe worauf sich „normalerweise" bezieht (z.B. auf ausgeführte Handlungen oder wie man sich Handlungen üblicherweise vorstellt). Daher haben wir dies zu „in Wirklichkeit" geändert. Dies wurde von den Versuchspersonen als eindeutig empfunden.

Anhang B

In Tabelle B1 sind die Faktorladungen aller Items der deutschen und englischen Version dargestellt.

Tabelle B1. Faktorladungen der Items im MTMM Ansatz des deutschen VMIQ-2 und des englischen VMIQ-2

	Deutsch			Englisch		
Item	EVI	IVI	KIN	EVI	IVI	KIN
EVI1	0.75	0	0	0.74	0	0
EVI2	0.75	0	0	0.74	0	0
EVI3	0.65	0	0	0.78	0	0
EVI4	0.72	0	0	0.78	0	0
EVI5	0.73	0	0	0.75	0	0
EVI6	0.67	0	0	0.75	0	0
EVI7	0.7	0	0	0.74	0	0
EVI8	0.61	0	0	0.73	0	0
EVI9	0.69	0	0	0.72	0	0
EVI10	0.64	0	0	0.71	0	0
EVI11	0.57	0	0	0.68	0	0
EVI12	0.61	0	0	0.72	0	0
IVI1	0	0.71	0	0	0.73	0
IVI2	0	0.69	0	0	0.74	0
IVI3	0	0.64	0	0	0.78	0
IVI4	0	0.73	0	0	0.76	0
IVI5	0	0.68	0	0	0.76	0
IVI6	0	0.69	0	0	0.7	0
IVI7	0	0.65	0	0	0.73	0
IVI8	0	0.56	0	0	0.67	0
IVI9	0	0.65	0	0	0.7	0
IVI10	0	0.6	0	0	0.67	0
IVI11	0	0.58	0	0	0.72	0
IVI12	0	0.58	0	0	0.66	0
KIN1	0	0	0.73	0	0	0.72
KIN2	0	0	0.74	0	0	0.77
KIN3	0	0	0.7	0	0	0.77
KIN4	0	0	0.7	0	0	0.78
KIN5	0	0	0.71	0	0	0.75
KIN6	0	0	0.68	0	0	0.67
KIN7	0	0	0.64	0	0	0.73
KIN8	0	0	0.65	0	0	0.69
KIN9	0	0	0.67	0	0	0.72
KIN10	0	0	0.67	0	0	0.68
KIN11	0	0	0.63	0	0	0.6
KIN12	0	0	0.6	0	0	0.62

© 2019 Hogrefe Verlag. Veröffentlicht als Hogrefe OpenMind-Artikel unter der Lizenz CC BY 4.0 (https://creativecommons.org/licenses/by/4.0)
